# Are Activity Wrist-Worn Devices Accurate for Determining Heart Rate during Intense Exercise?

**DOI:** 10.3390/bioengineering10020254

**Published:** 2023-02-15

**Authors:** Pilar Martín-Escudero, Ana María Cabanas, María Luisa Dotor-Castilla, Mercedes Galindo-Canales, Francisco Miguel-Tobal, Cristina Fernández-Pérez, Manuel Fuentes-Ferrer, Romano Giannetti

**Affiliations:** 1 Professional Medical School of Physical Education and Sport, Faculty of Medicine, Universidad Complutense de Madrid, 28040 Madrid, Spain; 2 Departamento de Física, FACI, Universidad de Tarapacá, Arica 1010069, Chile; 3 Instituto de Micro y Nanotecnología, IMN-CNM, CSIC (CEI UAM+CSIC), 28760 Tres Cantos, Spain; 4 Servicio de Medicina Preventiva Complejo Hospitalario de Santiago de Compostela, Instituto de Investigación Sanitaria de Santiago, 15706 Santiago de Compostela, Spain; 5 Unidad de Investigación, Hospital Universitario Nuestra Señora de Candelaria, 38010 Santa Cruz de Tenerife, Spain; 6 IIT, Institute of Technology Research, Universidad Pontificia Comillas, 28015 Madrid, Spain

**Keywords:** heart rate, wearables, physical exertion, exercise prescription, digital health, monitoring, photopletismography, accuracy, medical devices

## Abstract

The market for wrist-worn devices is growing at previously unheard-of speeds. A consequence of their fast commercialization is a lack of adequate studies testing their accuracy on varied populations and pursuits. To provide an understanding of wearable sensors for sports medicine, the present study examined heart rate (HR) measurements of four popular wrist-worn devices, the (Fitbit Charge (FB), Apple Watch (AW), Tomtom runner Cardio (TT), and Samsung G2 (G2)), and compared them with gold standard measurements derived by continuous electrocardiogram examination (ECG). Eight athletes participated in a comparative study undergoing maximal stress testing on a cycle ergometer or a treadmill. We analyzed 1,286 simultaneous HR data pairs between the tested devices and the ECG. The four devices were reasonably accurate at the lowest activity level. However, at higher levels of exercise intensity the FB and G2 tended to underestimate HR values during intense physical effort, while the TT and AW devices were fairly reliable. Our results suggest that HR estimations should be considered cautiously at specific intensities. Indeed, an effective intervention is required to register accurate HR readings at high-intensity levels (above 150 bpm). It is important to consider that even though none of these devices are certified or sold as medical or safety devices, researchers must nonetheless evaluate wrist-worn wearable technology in order to fully understand how HR affects psychological and physical health, especially under conditions of more intense exercise.

## 1. Introduction

Currently, wearable technology applied to biomedical data control in sports medicine is widespread among different types of users (e.g., athletes, patients, and people who practice sports or exercise), and is continuously growing [1,2,3,4,5,6,7,8,9,10,11,12,13]. Exercise as medicine is a global health initiative encouraging physicians and other healthcare professionals to include physical activity evaluation and training programs in every patient visit [14]. In this framework, wearable technology applied to health and fitness has been confirmed as a ubiquitous technology that helps to enhance performance and prevent injury [3,7,8,12,15,16]. Indeed, Google Trends 2020 reports with respect to the consumers’ increasing interest in wearable devices indicate that they are probably attracted by a desire to improve their quality of life [17].

Electronic medical devices based on light emitters (LED) have been on the market for several decades. Their use for medical care was first proposed in the late 1990s [18]. Biomedical data monitoring in daily life unquestionably has the potential to prevent and predict diseases with almost no inconvenience to the user [3,6,19]. As a matter of fact, only within these last two decades have common portable devices been developed to monitor health in daily life, providing the opportunity for different types of users to self-monitor their own physical activity [1,2,5,6,7,9,12,13,19,20,21,22]. Thompson’s global electronic survey, which determines health and fitness trends over the past the year, indicates that the use of wearable technology for sports and health medicine has been the number one trend beginning with its first introduction in the survey in 2016, except for 2018 when it was third and 2021 when it was second [1,14]. Ten years ago, a study conducted in the United States indicated that 69% of the adult population wore activity wristbands for personal monitoring [23]. Other reports predicted that sales of activity wristbands for sport would exceed USD 5 billion by 2019 [24] and that the number of connected wearable devices would reach more than one billion by 2022 [25].

Nevertheless, there are many inaccurate and inconclusive wearable devices on the market that purport to monitor activity, such as smart watches, heart rate monitors, GPS tracking devices, activity trackers, and smart glasses [1,14,21]. Indeed, recent studies recommend caution when interpreting metrics reported from consumer-wearable devices [10]. Earlier models of wearable devices relied solely on movement sensors such as accelerometers to estimate energy expenditure [26]. However, novel devices include photoplethysmogram (PPG) signals [27,28,29], which allow estimation of other data, such as biomedical data, and have improved accuracy when estimating energy expenditure [30,31]. This technology has been applied to wrist devices due to improvements in the miniaturization of the processing hardware (to convert raw signals in real-time into interpretable data) and to longer battery life.

The heart rate (HR), which increases with exertion, can be used to measure the intensity of an exercise session or to correlate with the maximum oxygen uptake $VO_{2,max}$ as an indicator of aerobic endurance [32,33]; $VO_{2,max}$ represents the maximum capacity of the human body to consume oxygen during activity. Hence, it is the most accurate and simple indicator for determining aerobic work capability [34]. A lower HR for a given exertion indicates greater cardiorespiratory and muscular fitness. Therefore, HR frequencies can be used to prescribe exercise, either as a percentage of an individual’s maximum HR reserve or based on a certain threshold determined through different mathematical algorithms [35,36,37]. In addition, HR variations serve as important markers for training adaptations and cardiovascular fitness assessment [18,38,39,40].

Although HR was first identified as an index of metabolic rate [41], nowadays HR estimation is a valuable indicator of physiological adaptation and intensity of effort [42,43,44]. In clinical settings, HR is frequently monitored via electrocardiogram (ECG), which uses electrodes on the chest to detect the heart’s repolarization and depolarization, or by photoplethysmography, such as in a pulse oximeter [10,45]. Heart Rate Variability (HRV) measures the interval between successive heartbeats [46], and is correlated with autonomic nervous system function [10]. In contrast to a diseased heart, a healthy heart exhibits a degree of time oscillation between beats [47]. When comparing HRVs between exercise sessions, changes in HRV can help to determine training intensities and might indicate over-training or sickness [48,49,50].

In recent years, the development of new non-invasive methods and systems such as PPG, optical spectroscopy, and pulse oximetry have improved HR measurements during exercise [51,52]. Wrist-worn devices for activity and human resources monitoring have become increasingly popular, and can help motivate athletes or patients [53]. However, wearable technology faces several important limitations, some biologically inherent and others technological, that avert the proliferation of wearable medical devices, including the lack of high-quality validation studies at different intensity levels [3,13,15,19,42,43,54,55,56,57,58,59,60,61]. Although accuracy in HR measurement is acceptable in chest strap and electrode-based heart rate monitors, the accuracy of HR measurement in wrist wearables with PPG is uncertain [56,62,63,64,65,66]. The accuracy of any wearable medical device is increasingly relevant, as it can influence both medical decisions and patient outcomes [15,19,42,43,60]. In cardiac patients as well as other patients, after hospital discharge [67] an integral validation of these devices is vital in light of the need to monitor the patient’s cardiac rehabilitation through the recommended HR thresholds [68,69,70].

Furthermore, there are additional limitations for wrist-worn devices that could have negative effects. In steady-state aerobic exercises such as cycling, wrist-worn activity devices have proven to be reasonably accurate in estimating HR [63,71]. However, in non-steady exercises such as body weight lifting, CrossFit, and other high-intensity interval training (HIIT) exercises, accuracy is compromised [54], especially at HR frequencies higher than 150 bmp [72,73,74]. Furthermore, because these devices are typically worn on the nondominant wrist during these multiple forms of exercise, motion artifacts may introduce noise in the detected signal [27,75,76,77]. Hence, for fitness professionals who supervise users to monitor their physical activity it is crucial to evaluate the accuracy of these devices.

Consequently, considering the growing popularity of wearable devices with consumers and researchers [4,5,8,10,56,57], queries about their reliability are becoming of paramount importance, along with the need to assess their validity and accuracy for health and recreation purposes. Unlike clinically approved devices, further research is needed to evaluate these devices. Therefore, a deeper analysis of the data obtained through these new devices is urgent, as only 5% of these technologies have been formally validated [10,63,78,79,80].

The current investigation aims to analyze the accuracy of four of the most popular wrist-worn devices, Fitbit Charge (FB), Apple Watch (AW), Tomtom Runner Cardio (TT), and Samsung G2 (G2), as a function of different intensity levels during a maximum stress test performed either on a treadmill or a cycle ergometer. A second goal of this study is to determine whether these devices are suitable as medical devices for assessing exercise safety or user health. The rest of this paper is structured as follows. Section 2 describes the selection of the participants in Section 2.1, the study settings in Section 2.2, the tested heart rate devices in Section 2.3, and the statistical analysis in Section 2.4. The results and data analysis are presented in Section 3 and discussed in Section 4. Finally, Section 5 provides our conclusions based on the main findings from this research.

## 2. Methods

### 2.1. Participant Selection

The inclusion criteria for subject selection were: athletes aged 18 to 55 performing regular practice of a competitive sport in national and regional tournaments for at least two years prior to the study and not having any current physical limitations, medical conditions, or psychiatric conditions. All subjects trained two to four times a week between 1 and 3 h/day. The volunteers maintained this sports practice until the day before the present study was carried out. Prior to admittance to the study, all subjects were evaluated for their cardiovascular health. None of the volunteers reported any respiratory or cardiac disease, presenting average spirometric values. Eight active and healthy athletes (85.7% male) who met the aforementioned inclusion criteria, volunteered to participate in this study. The athletes performed an incremental exercise test on a treadmill or a cycle ergometer while wearing two devices at the same time, one on each wrist, while electrocardiography data were recorded.

### 2.2. Study Setting

The exercise tests were performed in the Physiology Laboratory at the Professional School of Sports Medicine of the Faculty of Medicine, Universidad Complutense de Madrid, Spain. In conformity with the review policy statement, the experimental protocol was approved by the Ethics Committee of the Hospital Clinico San Carlos, Madrid (HCSC) (no: 16/123-E) and conducted according to the Helsinki Declaration. All subjects provided their written informed consent to participate after the procedure and the study risks had been explained to them.

The maximal stress tests were carried out either on a treadmill ergometer with an incremental protocol (fixed slope of 1%) reaching a final speed of 16 km/h, or on a cycle ergometer with an incremental protocol of 25 W/min reaching a power that oscillated between 250 and 350 W, with all the athletes reaching HR frequencies higher than 150 bpm.

**Figure 1:**
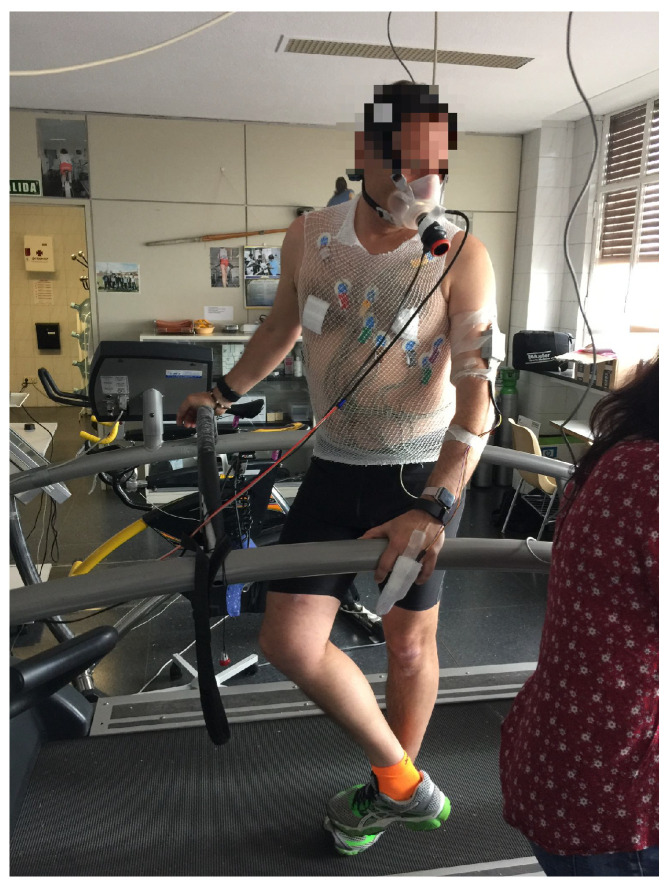
Performance athlete during the exercise stress test on the treadmill. The HR device is placed on the wrist, the electrodes are placed for electrocardiographic recording, and the mouthpiece is for the gas flow analyzer.

HR time series were extracted from the devices and compared with the HR data obtained from the electrocardiogram. During the athletes’ preparation, twelve ECG electrodes were placed for the 12-lead ECG reading. The area was first prepared by shaving and alcohol sterilization to ensure a correct electrode position while wearing a tubular mesh top. Subsequently, blood pressure was taken to establish a baseline measurement and electrocardiographic readings were taken at rest in supine and standing positions. At the beginning of the test, time and data were synchronized among the electrocardiogram and the wrist-worn devices. Parameter readings and measurements during the stress test were collected every 10 seconds. In addition, a mask was placed over the athlete’s nose and mouth in order to prevent air leakage and properly analyze expired gases, as shown in Figure 1.

### 2.3. HR Measuring Devices

For this study, four of the most popular wrist-worn devices (Fitbit Charge (Fitbit, CA, USA), Apple Watch (Apple, CA, USA), Tomtom runner Cardio (Tomtom, Amsterdam, The Netherlands), Gear S2 (Samsung, Suwon, Republic of Korea)) were selected to verify the HR accuracy because of their commercial availability, cost-effectively, light-weight, and popularity. The accuracy of these four wrist devices during a maximal exercise stress test was evaluated using a Norav 12-lead exercise electrocardiographic (ECG) recorder as the gold standard. The participants wore two different devices on each wrist, and wore the ECG at the same time. The data extraction procedure and characteristics of each device are specified in the following subsections.

#### 2.3.1. Tomtom Runner Cardio (TT)

Exercise data extraction was straightforward using the standard tcx format from Garmin. The data recording interval was one second.

#### 2.3.2. FitBit (FB)

This device featured the option of exporting exercise data in the standard tcx format from Garmin. The data recording interval was five seconds.

#### 2.3.3. Apple Watch (AW)

This device did not offer a data export function from a specific exercise session to a computer; extraction is only possible globally. The device produces a folder containing a compressed XML file with the complete measurement history (the file size can easily exceed 200 MB). Although the document’s format is specific to the device’s brand, a simple parser was written to select XML tags related to the pairs of data (time, HR frequency) during the stress test. The data recording interval was five seconds.

#### 2.3.4. Gear S2 (G2)

This device did not offer the possibility of extracting the data to a file that could be directly exported. The method for extracting the HR time series lay in producing screenshots of the S-Health application and exporting the data in numerical form using an ad hoc algorithm. The data recording interval was five seconds.

### 2.4. Statistical Analysis

Following the British Standards Institution 2019 standards for medical electrical equipment, the accuracy root mean square ($A_{rms}$) difference between the HR values of the device and the HR values of the ECG as a gold standard was calculated [81]. The $A_{rms}$ includes a combination of a standard systematic error or bias component and a random component, providing a single number as a measurement of both bias and precision. Therefore, the $A_{rms}$ statistic is usually evaluated when the overall accuracy of a device is tested [82].

(1) $$A_{rms}=\sqrt{\frac{\sum_{i=1}^{N}{\left(HR_{testeddevice}-HR_{ECG}\right)}^{2}}{N}}$$

Furthermore, the difference in accuracy between each device and the gold standard ECG was assessed by determining the interclass correlation coefficients (ICC) and their 95% CI, then constructing Bland–Altman graphs. Mean overall scores were compared between the devices and ECG using Student’s t-test for paired samples. For each test, the level of significance was set at 5%. Statistical analysis was performed using the SPSS 15.0 package. Quantitative variables are presented with their means and standard deviations (SD).

Lastly, the absolute percent errors (APE) with respect to ECG were calculated to construct ordered boxplots stratified by HR. The APE is provided by

(2) $$\%Error=\frac{HR_{testeddevice}-HR_{ECG}}{HR_{ECG}}\cdot 100$$

The Tukey outlier detection rule was used to find any extreme outliers in APE values for each device and metric combination [83]. Spearman’s rank correlation (R) was calculated for each device, with the results provided in the Supplementary Material, Figure S2.

## 3. Results

The tested devices, athletes’ sports, and anthropometric characteristics such as age, size, weight, height, and body mass index (BMI) are presented in Table 1. Based on the BMI and $VO_{2,max}$, respectively, all eight volunteers (seven men and one woman) were classified as having normal weight and good physical fitness condition [84].

**Figure 2:**
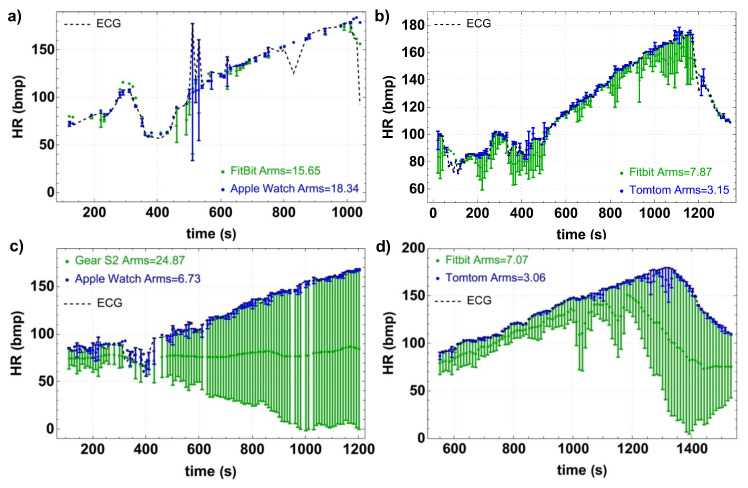
Wristband measurements for four athletes compared to the reference ECG data in the two different tests: treadmill (top panels, (**a**,**b**)) and cycle ergometer (bottom panels, (**c**,**d**)). The black line corresponds to the ECG data, while the blue and green lines correspond to the tested devices. The error bars denote differences between the ECG data and the tested device.

Figure 2 represents the HR measurements provided by the wristband for four athletes compared to the reference ECG data in the two different tests, treadmill (top panels, (a) and (b)) and cycle ergometer (bottom panels, (c) and (d)) at all exercise intensities. In all the graphs, the black line corresponds to the reference curve related to the ECG data, which is considered the benchmark of the test, while the blue and green lines correspond to the tested devices. The differences with the ECG data are marked by error bars. As can be seen, the HR rises steadily according to the athlete’s increasing effort intensity during the test. The accuracy root-mean-square ($A_{rms}$) was calculated for each device. The TT and AW devices have the lowest value, while the G2 has the highest value, which indicates worse accuracy.

All devices show high sensitivity to motion artifacts and fail to follow accurate HR when the athletes reach levels of maximum effort (higher HR). Motion artifacts are more perceptible in the treadmill test than in the cycle ergometer test due to the athlete’s movements. However, a high variability exists between the devices under the same conditions, i.e., the same type of test (cycle ergometer or treadmill) and HR. A comparison of the performance of the devices for the eight volunteers and a deeper analysis of the Spearman’s rank correlation (R) can be found in the Supplementary Materials, Figures S1 and S2.

**Figure 3:**
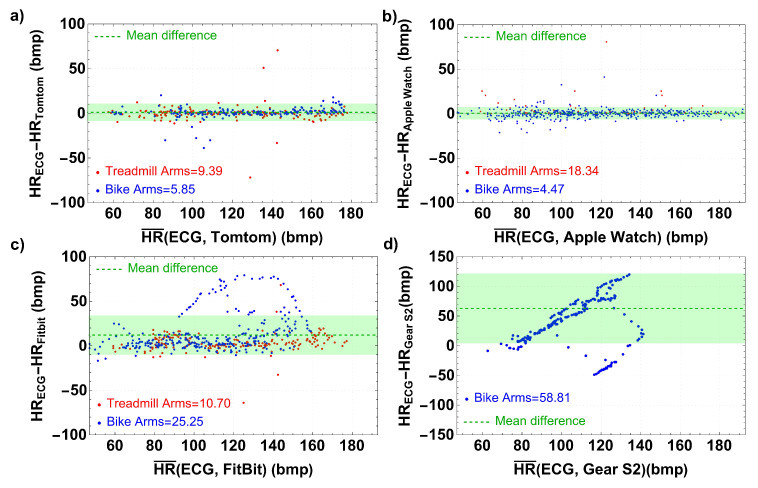
Bland–Altman diagrams of successive differences showing heart rate measurements for the four devices. The mean heart rate is shown on the x-axis and the difference between the specified device and the ECG rate is shown on the y-axis. Red dots correspond to the test on the treadmill and blue dots correspond to the test on the cycle ergometer. The green areas indicate the limits of the agreement. Panel (**a**) compares the TT runner and the ECG, Panel (**b**) compares the AW and the ECG, Panel (**c**) compares the FB Charge and the ECG, and panel (**d**) compares the G2 and the ECG.

All available data from each instrument were processed in Bland–Altman form in order to obtain a more global view of the devices’ performance. Data was reduced to measurements every 10 s (the available ECG data rate) by averaging or interpolating the extracted data from the four devices. The results provided by the four tested devices at all exercise intensities are shown in Figure 3. The blue dots correspond to the data extracted from the cycle ergometer tests, while the red dots correspond to the tests on the treadmill. The green areas display the limits of agreements for each device, and the dashed green line corresponds to the mean difference. The $A_{rms}$ values are specified for each test and device.

High variability and significant inaccuracies between the ECG and the device HR measurements at high exercise intensities were observed among the different tests. For this reason, we performed a detailed statistical study based on different HR ranges (less than 110 bpm, from 110 to 150 bpm, and greater than 150 bpm). Table 2 shows the differences in the paired means between the ECG and each device for the whole sample and stratified by HR range (≤100 bpm, 110–150 bpm y > 150 bpm). We analyzed 1,286 simultaneous HR data pairs between the four tested devices and the ECG used as the reference standard. There were a total of 321 pairs from the TT runner, 440 from the AW, 377 from the FB, and 148 from the G2. The mean (SD) of each device, mean (SD) of the difference between the tested device measurement and the reference standard, mean relative difference (SD; %), mean absolute difference (SD; %), correlation between the measures, and interclass correlation coefficients (ICC) were determined, along with their 95% CIs.

**Figure 4:**
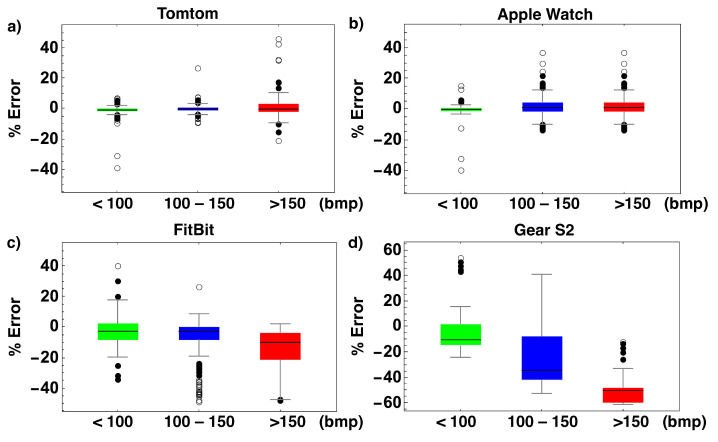
Box plot visualization of the tested devices’ absolute percent error (APE). The green boxes correspond to HR measurements below 100 bmp, the blue boxes correspond to HR measurements between 100-150 bmp, and the red boxes correspond to HR measurements greater than 150 bmp. (**a**) TT, (**b**) AW, (**c**) FB, and (**d**) G2.

Lastly, device boxplots stratified by HR intervals were performed to assist with visualization and analysis. Following Equation (2), Figure 4 shows the absolute percent errors (APE) for each device. The box limits show the range of 50% of the data, with a center black line designating the median value. The lines extending from each box represent the range of the remaining data, with dots placed there to represent outlier values. The empty dots refer to the most distant outliers. The green boxes correspond to HR measurements below 100 bmp, the blue boxes correspond to HR measurements between 100–150 bmp, and the red boxes correspond to HR measurements greater than 150 bmp.

## 4. Discussion

This current investigation examined how effectively four popular wrist-worn activity monitors (Fitbit Charge, Apple Watch, Tomtom Runner Cardio, and Gear S2) estimated HR throughout a maximal test performed either on a treadmill or a cycle ergometer. We found reasonable accuracy in HR estimation for two of these devices (AW and TT), especially at lower-intensity exercises, which is consistent with earlier studies [31,43,62,69,85]. Our findings indicate a positive difference in averages between the ECG and each device. Therefore, the tested devices tend to underestimate HR concerning the ECG, which is more noticeable in the case of the lower accuracy devices, namely, the FB and the G2. These results concur with other previous studies that examined HR estimations across various devices [63,71], although these studies only carried out monitoring after HR had stabilized under steady-state settings. According to another assessment, HR readings are typically more accurate on a cycle ergometer or at rest than on a treadmill [86]. Indeed, exercises involving an unstable wrist, such as those performed on elliptical machines or when walking or running provide less accurate HR readings than exercises involving a stable wrist, such as the cycle ergometer [87,88].

Although the lowest $A_{rms}$ when measuring HR were observed for the TT and the AW, as shown in panels (a) and (b) of Figure 3, significant variability between the different tests can be observed. Motion artifacts such as oscillation or arm movements are more visible for HR frequencies above 130 bmp. Indeed, in the case of AW, considerable differences appear for the treadmill test due to motion artifacts (see the red dots). On the contrary, for FB the appearance of a large cluster of blue points in the top part of panel (c) indicates that the $A_{rms}$ is higher for the cycle ergometer than the treadmill. Similar behavior was observed in a comparative study of the Fitbit Charge 2 and Garmin Vivosmart HR. Here, a significantly lower relative error was found for activities with repetitive motion of the upper torso compared to activities with no repetitive motion of the upper torso, such as the cycle ergometer test [54]. These unexpected differences could be a consequence of the configuration of the cycle ergometer, which has a magnetic brake that can interfere with non-shielded devices [89,90]. The G2 observed a remarkably high bias (mean difference) at almost all levels of exercise intensity (see panel (d) of Figure 3), which is in agreement with the reported results of a previous study [63]

In addition, motion artifacts present one of the most challenging problems for HR estimations under extreme activity settings [91,92]. Prior studies have revealed a lack of accuracy of PPG sensors when determining HR during activities involving significant physical exertion or repetitive contractions of the muscles [27,52,87,93,94], particularly above 150 bpm [74,95]. PPG signals can be obstructed, resulting in poor data quality due to a reduction in contact between the device’s PPG sensor and the skin during activities involving prolonged muscle contractions or more intense workouts [27,94]. In addition, according to Parak et al., the type of sensor and the position in which the device is worn are significant factors determining the accuracy [96].

Within the last decades, effective algorithms to improve the quality of the signal in the presence of motion artifacts for exercises performed above 150 bpm have been developed [87,91,97,98,99,100], for instance, by processing context information using additional on-body sensors and light sources [101,102], adaptive noise cancellation using accelerometers as a noise reference [103], adaptive noise cancellation using an integrated PPG sensor [104], deep learning methods [105], and techniques based on spectral analysis, such as traditional fast Fourier transform (FFT) [27].

The majority of previous studies carried out on the determination of HR on wrist-worn devices have shown limited accuracy [85,106,107,108], typically with measurements that may be somewhat understated [76,109,110,111]. A number of studies have attempted to correlate wearable HR measurements by PPG with those from a reference ECG signal as a gold standard [20,51,85,93,109]. Indeed, Boudreaux et al. simultaneously determined the accuracy of eight wearable devices, six wrist-worn, one chest-worn, and one ear-worn, during a graded cycling exercise test and during a structured resistance exercise regimen [111]. They found a significant underestimation of HR as exercise intensity increased across all devices; however, none of these studies analyzed accuracy according to HR stratification.

A study of FB and AW for very light, light, moderate, vigorous, and very vigorous intensities based on ECG-measured HR showed that the AW had the lowest relative error rate compared to FB at all exercise intensities; however, the accuracy of both devices was reduced as exercise intensity increased [112]. A study evaluating the accuracy of the Polar M600 optical heart rate monitor during various physical activities reported the highest HR percent accuracy during cycle intervals and the lowest during circuit weight training. In addition, there was a tendency toward HR underestimation as intensity increased and toward overestimation when intensity decreased. The accuracy was higher during periods of steady-state cycling, walking, jogging, and running, though less accurate as intensity increased [113].

In this context, [62] tested the accuracy of six types of wearable devices on 50 volunteers walking or running on a treadmill. In their study, the TT showed the best accuracy while the FB showed the poorest, which is similar to the results we report here. According to a recent systematic review of studies of various models of FB devices against reference measures of energy expenditure, heart rate, and steps, FB devices are likely to underestimate heart rate. However, this underestimation can, on average, be acceptable for steps and heart rate [114].

Moreover, under conditions of intense physical activity the accuracy of heart rate measurement is significantly decreased [64,110,115]. Studies have yet to critically compare devices with a gold standard method approved by the FDA. Indeed, the manufacturers have yet to propose a robust validation system for these devices [61,63,68]. The type of exercise and the conditions in which the exercise is performed influence the reproducibility of HR measurement by these devices [74,116,117]. Although there are many studies about accuracy in the determination of HR in wrist-worn devices during rest [118,119], as well as in different physical activities (sitting, standing, walking slowly, walking fast, running, cycling, etc.) [49,63,64,74,76,85,106,110,120,121,122,123,124,125,126,127,128,129,130,131], to the best of our knowledge there has been no research into the reliability of these specific devices focused on heart rate ranges compared to conventional ECG results.

In this framework, one of our goals was to determine a threshold HR value at which motion artifacts become significant. For our analysis based on stratified HR ranges, see Table 2; it can be seen that the TT and AW devices present insignificant differences in means concerning the ECG in the intervals <100 HR, with ICC = 0.683 and ICC = 0.799, respectively. Between 100–150 HR, they show a higher degree of accuracy, with good or excellent accuracy when ICC = 0.938 and ICC = 0.930, respectively. It is somewhat surprising that the ICC is lower for HR < 100 bmp than in the 100–150 HR interval. This fact could be attributed to distortion of the PPG signal or delays in the device response time to HR variations. Indeed, as Iyriboz et al. assumed, during heavy exercise for HR > 155 bmp the oscillations of the pulse pressure waveform are distorted in a way that interferes with the PPG signal [74].

Figure 3 shows that the highest differences for TT start at 128 bmp for the treadmill test, with an $A_{rms}$ = 9.39, while for the cycle ergometer test the outlier values appear at HRs greater than 150 bmp, with an $A_{rms}$ = 5.85. In the case of the AW, although there are isolated dots for HR between 100 and 120 bmp for both tests, the $A_{rms}$ = 18.34 for the treadmill test, while for the cycle ergometer we obtain a low $A_{rms}$ = 4.47. In addition, as the intensity increases in the HR ≥ 150 bmp range the level of accuracy is moderate, with ICC =0.528 and ICC =0.729, respectively, maintaining minor differences in the means concerning the ECG. These results corroborate those of Boudreaux et al. [111] and Wang et al. [69], who reported that the AW was highly accurate in measuring heart rate during graded exercise cycling and various aerobic activities, respectively.

Furthermore, for the FB and G2 the differences between the HR averages and the ECG data increase significantly as the HR increases, showing a poor level of accuracy, especially for HR ≥ 150 HR, for which the ICCs are 0.019 and −0.024, both with *p* values less than 0.001. Panel (c) of Figure 3 shows a high $A_{rms}$ = 25.25 for the FB device in the cycle ergometer test at frequencies between 100–150 bmp, with a low ICC =0.148. In contrast, a lower $A_{rms}$ = 10.70 was estimated for the treadmill test. Both of these results are consistent with the study by Reddy et al. [54]. Lastly, the performance of the G2 provides inaccurate measurements, with ICC <0.005 in the three studied HR ranges. This low performance may have resulted from improper development of the HR determination algorithms.

Figure 4 shows a box plot representing the APE for each of the four devices we evaluated stratified by HR intervals. As can be observed, the TT and the AW display lower APE values for HRs below 100 bmp and between 100 and 150 bmp, while their accuracy is reasonable for frequencies above 150 bmp. The FB device offers an acceptable level of accuracy for HR values below 100 bmp and between 100 and 150 bmp; however, the precision is unreliable for frequencies above 150 bmp. The G2 has strong APE values across all three examined ranges. Figures S1 and S2 of the Supplementary Material show the threshold values at which motion artifacts become to be perceptible for all four devices.

To the best of our knowledge, this study is the first to assess the reliability of these particular wrist-worn wearable devices (FB, TT, AW, G”) based on various HR ranges while examining the impact of exercise intensity. Our findings demonstrate that HR accuracy is markedly compromised across all devices as exercise intensity increases. Therefore, our stratified and correlated study should be taken into account when prescribing exercise, especially for people with underlying heart disease.

### 4.1. Main Implications and Future Perspectives

The aim of this study was to assess the accuracy of the chosen wearables as well as to determine whether these devices are suitable as medical devices to assess exercise safety or user health. It is important to increase the precision of the equipment that measures medical parameters in order to assist athletes and patients with heart disease and lower the risk of harm when exercising. It is essential to keep in mind that even if none of these devices is certified or sold as a medical or safety device, their use is widespread within the population, particularly in occasional and non-professional athletes [1,14,21]. In addition, not all wrist activity monitors are made to exact requirements. A number of them have demonstrated unreliable accuracy when used for various activities and exercise settings, including extreme physical activity [10,23,63,78,79,80]. It is worth mentioning that this behavior can result in inaccurate or underestimated readings of the relevant physical effort level, which can cause harmful behavior for unaware users.

Indeed, in order to fully understand how HR affects psychological and physical health, future research to evaluate wrist-worn wearable technology is needed in order to maximize the usefulness of new technologies, clarify the accuracy of physiological data under conditions of more intense exercise, and clearly resolve researchers’ claims to satisfy the FDA-approved gold standard. Regulatory standards must be prepared to ensure the process of accurate evidence accumulation. Considering that companies rarely fully validate new wearable models, it is important to use caution when comparing our findings to earlier models, as it is unknown whether the sensors or algorithms have changed.

### 4.2. Strengths and Limitations of This Study

This research has the following strengths over prior studies. First, this study simultaneously assessed the HR accuracy of four popular wrist-worn activity monitors using the most recent estimation techniques. For the calibration and validity of wrist-worn activity monitoring devices, the use of “unit calibration” allows the signals to be appropriately monitored. In these studies, it is imperative to evaluate different parameters such as exercise intensity [129,130], different skin pigmentation [132], sex, age range, and fitness condition [133]. Second, we analyzed the absolute percent error for different HR ranges in order to determine the frequency range at which motion artifacts become noticeable. In addition, a stress test was performed on two different ergometers, namely, a cycle ergometer and a treadmill. Third, participants in this study received in-depth education and training, meaning that they were already familiar with how the devices worked. Fourth, utilizing an ECG as the gold standard was a sensible decision that prevented system error resulting from using instrument measurements. Lastly, for the analysis of the extracted data, we used a rigorous statistical methodology, the Bland–Altman method, which is considered the most appropriate statistical method for evaluating the measurement of biomedical variables [134]. Many investigations often engage in inappropriately analysis [68] by using correlation coefficients [115]. This statistical methodology is suitable for studying different HR ranges and the variance according to these HR ranges [45].

The study does, however, have certain limitations. First, the monitoring data were collected from participants in a laboratory under controlled conditions; therefore, the outcomes might not accurately mirror those in real-world settings. The amount of random and incidental error resulting from measurements obtained in the subject’s natural state of life can be significantly reduced by imposing specific constraints on the subject’s activity settings. Second, because reduced PPG accuracy is linked to increased wearable movement [135], there are various significant parameters that have been demonstrated to affect HR accuracy, including wrist placement, wrist circumference, device tightness, dominant vs. non-dominant hand use, and degree of wrist movement [109,136]. Lastly, only one cross-sectional measurement was made for this study on seven male and one female volunteer athletes of different ages, BMIs, and $VO_{2,max}$ with both light and dark skin. No additional longitudinal measures were made. The sample size for female participants (1) and those with dark skin (1) were not large enough to reach statistically significant conclusions, as described in other studies [120,132]. However, these limited sample sizes was partially offset by the simultaneous HR measurements made with a variety of wearable devices.

## 5. Conclusions

The main goal of the current study was to evaluate the performance and accuracy of four commercially available wrist-worn wearables for monitoring HR at various activity levels. Our results show that as exercise intensity increases there is a higher underestimation of HR across all devices. The FB and G2 have medical device features that do not meet the FDA-approved gold standard, and both are significantly incorrect above 150 bmp. Particularly significant is that in cardiac rehabilitation, where many of these devices are used, efficient intervention is needed to manage the intensity of physical exertion in order to produce accurate HR readings at high-intensity levels (above 150 bpm). On the contrary, the wrist-worn wearables developed by Apple and TT demonstrate the highest validity for monitoring HR during a physical activity at different levels. Therefore, the validity of exercise recommendations based on the heart rates measured by these devices is acceptable. However, because these devices are frequently used to collect physiological data in long-term medical investigations, more research must focus on varied populations and pursuits to validate these findings. Furthermore, manufacturers might find this comparison helpful in determining the general applicability of measurements provided by various vendors.

## Figures and Tables

**Table 1:** Anthropometric characteristics of the participants, their practiced sports, tested devices, and type of test. TM: treadmill; CE: cycle ergometer.

ID	Age (years)	Size (m)	Weight (Kg)	BMI (Kg/m${}^{2}$)	$VO_{2,max}$ (mL/kg/min)	Sport	Tested Devices	Test Type
00	40	1.85	81.6	23.84	52.10	Athletics	TT+FB	TM
01	39	1.69	69.9	24.47	57.42	Triathlon	TT+FB	CE
02	25	1.59	53	20.96	48.77	Athletics	AW+FB	CE
03	42	1.73	60	20.05	57.95	Athletics	AW+FB	CE
04	25	1.7	57.5	19.90	49.81	Soccer	AW+G2	CE
05	26	1.78	65.2	20.58	67.36	Athletics	AW+G2	CE
06	32	1.74	72	23,78	60.15	Cycling	AW+FB	CE
07	51	1.74	81	26.75	42.00	Athletics	AW+FB	TM
$\bar{X}$±SD	35 ± 9.53	1.73 ± 0.07	67.53 ± 10.55	22.54 ± 2.51	54.45 ± 7.88			

**Table 2:** Paired mean difference (ECG vs. device) and accuracy in the whole sample and stratified by HR range. SD: standard deviation; CI: confidence interval; HR: heart rate; ECG: electrocardiography; ICC: interclass correlation coefficient.

ECG vs. Device	N	Mean ECG (SD)	Mean Device (SD)	Mean Difference ECG-Device (CI 95%)	*p*	ICC (CI 95%)
**All Measurements**						
ECG vs. FB	377	127.5 (28.6)	115.5 (26.5)	11.9 (9.9; 14.0)	<0.001	0.675 (0.434; 0.798)
ECG vs. TT	321	134.9 (27.5)	133.9 (27.2)	1.1 (0.2; 1.9)	0.013	0.961 (0.952; 0.969)
ECG vs. AW	440	129.7 (29.2)	129.4 (29.5)	0.3 (−0.3; 1.0)	0.301	0.970 (0.96; 0.97)
EECG vs. G2	148	143.3 (25.4)	80.5 (14.2)	62.7 (58.1; 67.4)	<0.001	0.005 (−0.022; 0.040)
**Interval <100 HR**						
ECG vs. FB	117	94.3 (12.4)	89.7 (14.8)	4.5 (2.9; 6.1)	<0.001	0.746 (0.568; 0.844)
ECG vs. TT	75	98.2 (9.9)	98.3 (13.3)	−0.1 (−2.2; 2.1)	0.937	0.683 (0.540; 0.787)
ECG vs. AW	123	93.9 (12.3)	94.4 (14.8)	−0.5 (−2.0; 1.0)	0.514	0.799 (0.724; 0.855)
ECG vs. G2	16	103.6 (4.6)	76.8 (1.36)	26.7 (24.5–29.0)	<0.001	0.007 (−0.009; 0.052)
**Interval 100–150**						
ECG vs. FB	167	130.0 (11.6)	119.7 (18.6)	10.3 (7.2; 13.3)	<0.001	0.148 (0.004; 0.288)
ECG vs. TT	132	129.5 (11.07)	129.2 (11.6)	0.3 (−0.4; 0.9)	0.449	0.938 (0.914; 0.956)
ECG vs. AW	196	129.8 (11.6)	130.2 (11.9)	0.4 (−0.2; 1.0)	0.239	0.930 (0.908; 0.946)
ECG vs. G2	70	130.2 (11.9)	80.35 (15.3)	49.9 (45.8–53.9)	<0.001	0.030 (−0.030; 0.126)
**Interval >150 HR**						
ECG vs. FB	93	164.8 (8.7)	140.3 (21.9)	24.5 (18.5; 9.4)	<0.001	−0.019 (−0.111; 0.094)
ECG vs. TT	114	165.5 (8.4)	162.7 (10.4)	2.7 (1.0–4.4)	0.002	0.528 (0.374; 0.653)
ECG vs. AW	121	166 (10.7)	164.8 (13.8)	1.2(−0.4; 2.8)	0.146	0.729 (0.634; 0.803)
ECG vs. G2	62	168.3 (12.2)	81.7 (14.6)	86.6 (80.6–92.5)	<0.001	−0.024 (−0.035; 0.065)

## Data Availability

Not applicable.

## Supplementary Materials

The following supporting information can be downloaded at: https://www.mdpi.com/article/10.3390/bioengineering10020254/s1, Figure S1: Wristband measurements for the eight athletes compared to the reference ECG data in the two different tests: (a,b) treadmill, and (c–h) cycle ergometer. Figure S2: Scatter plots showing simultaneous HR measurements from ECG (x-axis) compared with each device (y-axis) in the different tests. The Spearman’s rank correlation (R) is shown for each case.

## Abbreviations

The following abbreviations are used in this manuscript:

HR	Heart Rate
HRV	Heart Rate Variability
bpm	beats per minute
ECG	electrocardiogram
FB	Fitbit Charge
AW	Apple Watch
TT	Tomtom Runner Cardio
G2	Samsung G2
LED	light emitting diode
PPG	photoplethysmogram
$A_{rms}$	accuracy root-mean-square
APE	absolute percent error
ICC	interclass correlation coefficient
SD	standard deviation
R	Spearman’s rank correlation
