# Supplementary material for: Are Activity Wrist-Worn Devices Accurate for Determining Heart Rate during Intense Exercise?
Source: Bioengineering (Basel). 2023 Feb 15;10(2):254. doi: 10.3390/bioengineering10020254 (PMC9952291; doi:10.3390/bioengineering10020254)
Supplement: Supplementary file 1 [file bioengineering-10-00254-s001.zip › bioengineering-2055859-supplementary.pdf]

bioengineering

MDPI

Article

Are Activity Wrist-Worn Devices Accurate for Determining Heart Rate during Intense Exercise?

Pilar Martín-Escudero [1], Ana María Cabanas [2,*], María Luisa Dotor-Castilla [3], Mercedes Galindo-Canales [1], Francisco Miguel-Tobal [1], Cristina Fernández-Pérez [4], Manuel Fuentes-Ferrer [5] and Romano Giannetti [6]

[1] Professional Medical School of Physical Education and Sport, Faculty of Medicine, Universidad Complutense de Madrid, 28040 Madrid, Spain
[2] Departamento de Física, FACI, Universidad de Tarapacá, Arica 1010069, Chile
[3] Instituto de Micro y Nanotecnología, IMN-CNM, CSIC (CEI UAM+CSIC), 28760 Tres Cantos, Spain
[4] Servicio de Medicina Preventiva Complejo Hospitalario de Santiago de Compostela, Instituto de Investigación Sanitaria de Santiago, 15706 Santiago de Compostela, Spain
[5] Unidad de Investigación, Hospital Universitario Nuestra Señora de Candelaria, 38010 Santa Cruz de Tenerife, Spain
[6] IIT, Institute of Technology Research, Universidad Pontificia Comillas, 28015 Madrid, Spain
* Correspondence: acabanas@academicos.uta.cl

check for updates

Citation: Martín-Escudero, P.; Cabanas, A.M.; Dotor-Castilla, M.L.; Galindo-Canales, M.; Miguel-Tobal, F.; Fernández-Pérez, C.; Fuentes-Ferrer, M.; Giannetti, R. Are Activity Wrist-Worn Devices Accurate for Determining Heart Rate during Intense Exercise?. *Bioengineering* **2023**, *10*, 254. https://doi.org/10.3390/bioengineering10020254

Academic Editors: Reuben Burch, Tony Luczak and Zachary M. Gillen

Received: 8 December 2022
Revised: 28 January 2023
Accepted: 30 January 2023
Published: 15 February 2023

Publisher's Note: MDPI stays neutral with regard to jurisdictional claims in published maps and institutional affiliations.

Copyright: © 2023 by the authors. Licensee MDPI, Basel, Switzerland. This article is an open access article distributed under the terms and conditions of the Creative Commons Attribution (CC BY) license (https://creativecommons.org/licenses/by/4.0/).

Abstract: The market for wrist-worn devices is growing at previously unheard-of speeds. A consequence of their fast commercialization is a lack of adequate studies testing their accuracy on varied populations and pursuits. To provide an understanding of wearable sensors for sports medicine, the present study examined heart rate (HR) measurements of four popular wrist-worn devices, the (Fitbit Charge (FB), Apple Watch (AW), Tomtom runner Cardio (TT), and Samsung G2 (G2)), and compared them with gold standard measurements derived by continuous electrocardiogram examination (ECG). Eight athletes participated in a comparative study undergoing maximal stress testing on a cycle ergometer or a treadmill. We analyzed 1,286 simultaneous HR data pairs between the tested devices and the ECG. The four devices were reasonably accurate at the lowest activity level. However, at higher levels of exercise intensity the FB and G2 tended to underestimate HR values during intense physical effort, while the TT and AW devices were fairly reliable. Our results suggest that HR estimations should be considered cautiously at specific intensities. Indeed, an effective intervention is required to register accurate HR readings at high-intensity levels (above 150 bpm). It is important to consider that even though none of these devices are certified or sold as medical or safety devices, researchers must nonetheless evaluate wrist-worn wearable technology in order to fully understand how HR affects psychological and physical health, especially under conditions of more intense exercise.

Keywords: heart rate; wearables; physical exertion; exercise prescription; digital health; monitoring; photopletismography; accuracy; medical devices

1. Supporting Information

Figure S1 represents the HR measurements provided by the four wrist-worn devices, the Fitbit Charge (FB), Apple Watch (AW), Tomtom runner Cardio (TT), and Samsung G2 (G2), for the eight athletes compared to the reference ECG data in the two different tests on the treadmill and the cycle ergometer at all exercise intensities. In all the graphs, the reference curve is the black one, related to the ECG data, which is considered the benchmark of the test. The blue and green lines correspond to the tested devices. The differences with the ECG data have been marked by error bars.

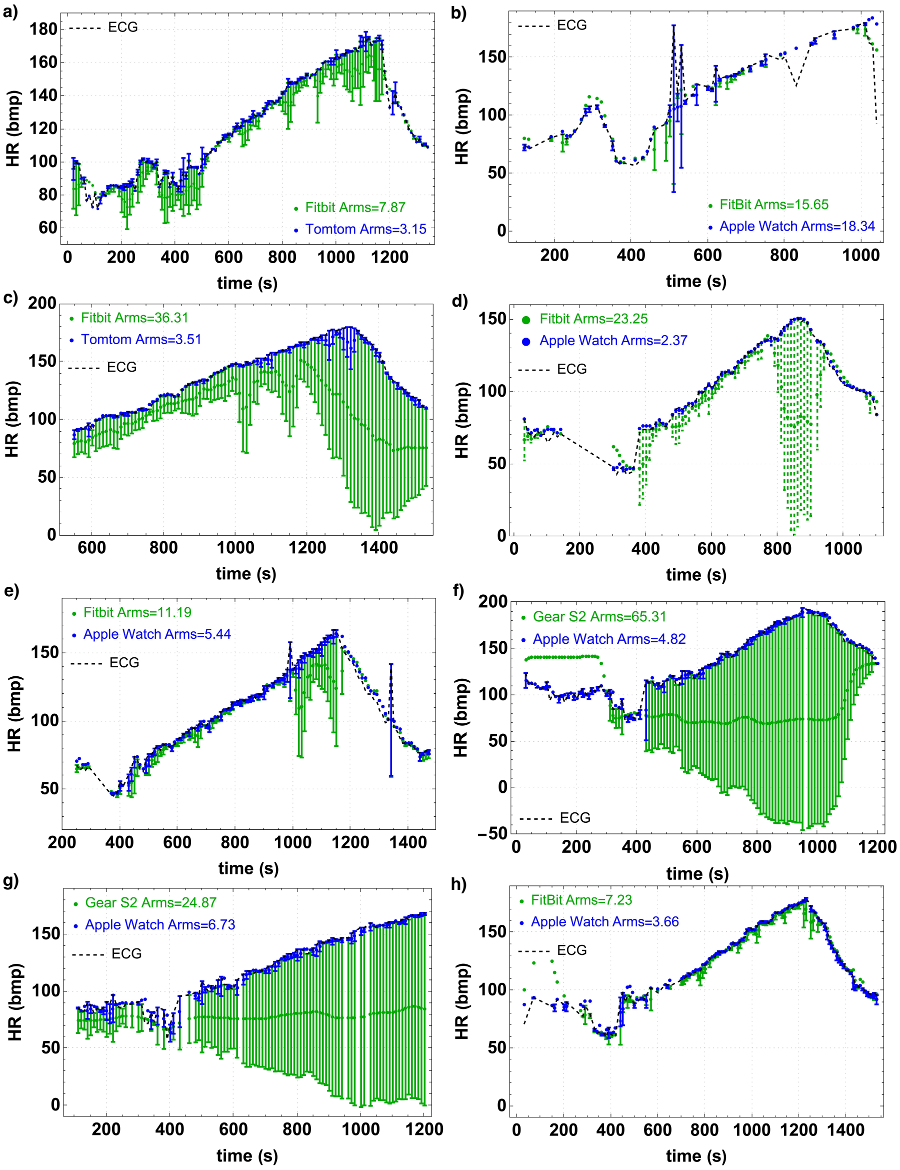

Figure S1. Wristband measurements for the 8 athletes compared to the reference ECG data in the two different tests: a) and b) treadmill; c), d), e), f), g), and h) cycle ergometer.

The HR rises steadily according to the athlete´s increasing effort intensity during the test. The accuracy root mean square (A_{rms}) is calculated for all devices. Panel a) shows the performance of the FB and the TT in the treadmill test for the volunteer 00. It can be seen that the accuracy of the TT is higher that the FB, with A_{rms} values of 3.15 and 7.87, respectively. Panel b) displays the performance of the FB and AW for volunteer 07. In this case, the accuracy of the FB is higher than the AW, with A_{rms} values of 15.65 and 18.34, respectively. This difference can be caused by motion artifacts and different positions of the device in the dominant or nondominant wrist during the test on the treadmill.

The performances of the FB and TT in the cycle ergometer test for volunteer 01 are shown in panel c). As in panel a), the accuracy of the TT is higher than FB, with A_{rms} values of 3.51 and 36.31, respectively. Let us also remark that, as other studies have reported, the FB device is quite sensitive to motion artifacts in a cycle ergometer test [1]. The AW and FB in the cycle ergometer test are compared in panels d), e), and h) for volunteers 02, 03, and 06. In all three cases, the AW demonstrated a higher accuracy than FB with lower A_{rms} values.

The AW and G2 are compared in panels f) and g) for volunteers 04 and 05. In both cases, the AW again demonstrated higher accuracy than the G2. The low performance of the G2 device is particulary remarkable, as it shows quite high values of the A_{rms}, especially at the end of the tests with higher HRs.

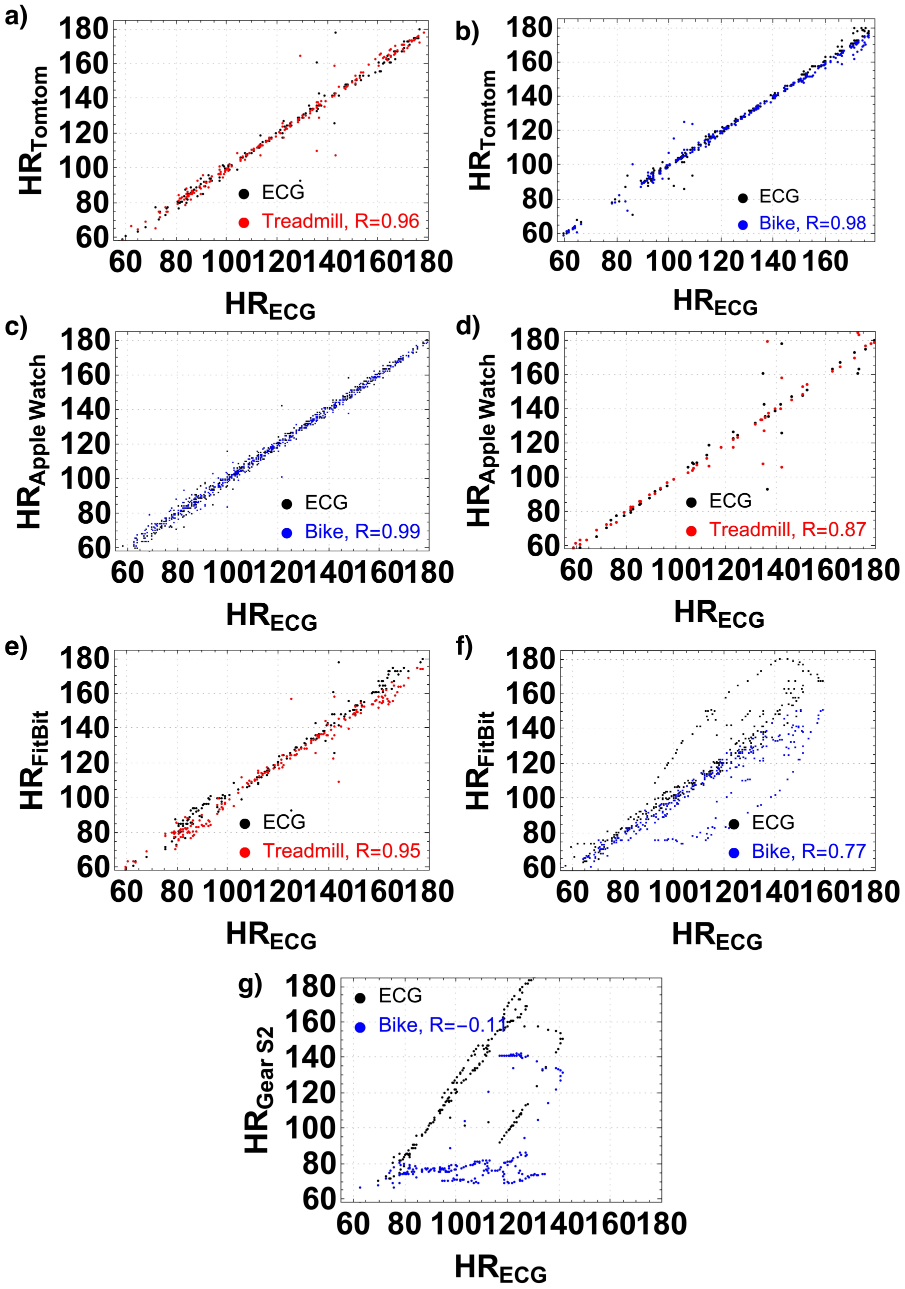

Figure S2. Scatterplots showing simultaneous HR measurements from ECG (x-axis) compared with each device (y-axis) in the different tests. The Spearman's rank correlation, R, is shown for each case.

For a deeper analysis of the differences between each device and the ECG, Figure S2 displays scatterplots showing simultaneous HR measurements from the ECG (x-axis) compared with each device (y-axis). In order to compare the different tests and devices, the Spearman's rank correlation, R, has been calculated for each device and test. As can be seen, the AW and TT present the highest R values for each test. The FB presents a low correlation with R=0.77 in the cycle ergometer test, while G2 shows a negative and very low correlation with R=-0.11.

Author Contributions: Conceptualization, P.M.-E., A.M.C., M.F.-F., C.F., and M.G.-C.; methodology, P.M.-E., M.T., and M.G.-C.; software, M.F.-F., R.G., and A.M.C.; validation, P.M.-E. and A.M.C.; formal analysis, A.M.C., M.F.-F, R.G., and C.F.-P.; investigation, P.M.-E., M.L.D.-C, M.G.-C., F.M.T., and C.F.-P.; resources, P.M.-E.; data curation, P.M.-E., A.M.C., and M.F.-F.; writing—original draft preparation, P.M.-E. and A.M.C.; writing—review and editing, P.M.-E. and A.M.C.; visualization, A.M.C.; supervision, P.M.-E.; project administration, P.M.-E.; funding acquisition, P.M.-E. All authors have read and agreed to the published version of the manuscript

Funding: This research was funded by the Higher Sports Council, Ministery Culture and Sport, FUNDER grant number (01/UPB10/07) "Estudio de la saturación de oxígeno, en deportistas mujeres de raza negra, durante la realiza-ción de pruebas de esfuerzo máximas" and FUNDER grant number: (01/UPB10/08) "Análisis y modelado de la relación entre el Electrocardiograma, fotopletismografía laser y parámetros ventilatorios durante la ejecución de pruebas de esfuerzo máximas en deportistas de ambos sexos".

Institutional Review Board Statement: The study was conducted according to the guidelines of the Declaration of Helsinki and approved by the Institutional Review Board (or Ethics Committee) of Hospital Clinico San Carlos (HCSC) no: 16/123-E.

Informed Consent Statement: Informed consent was obtained from all subjects involved in the study.

Acknowledgments: The authors would like to express their thanks to all of the athletes who participated in the study and to all the physicians from the Department of Sports Medicine and personnel from the Professional School of Sports Medicine. A.M.C and P.M. thank the support from ANID Project SA22I0178. A.M.C thanks the support from UTA-Project 4722-22 and from ANID Project SA77210039.

Conflicts of Interest: The authors declare no conflicts of interest.

Abbreviations

The following abbreviations are used in this manuscript:

HR	Heart Rate
bpm	beats per minute
ECG	electrocardiogram
FB	Fitbit Charge
AW	Apple Watch
TT	Tomtom runner Cardio
G2	Samsung G2
A_{rms}	accuracy root mean square
R	Spearmean's rank correlation

References

1. Reddy, R.K.; Pooni, R.; Zaharieva, D.P.; Senf, B.; El Youssef, J.; Dassau, E.; Doyle Iii, F.J.; Clements, M.A.; Rickels, M.R.; Patton, S.R.; et al. Accuracy of Wrist-Worn Activity Monitors During Common Daily Physical Activities and Types of Structured Exercise: Evaluation Study. *JMIR mHealth and uHealth* **2018**, *6*, e10338. https://doi.org/10.2196/10338.
